# Assessment of knowledge, attitude and practice towards post exposure prophylaxis for HIV among health care workers in Gondar, North West Ethiopia

**DOI:** 10.1186/1471-2458-13-508

**Published:** 2013-05-25

**Authors:** Biniam Mathewos, Wubet Birhan, Sebesbe Kinfe, Meaza Boru, Gemechu Tiruneh, Zelalem Addis, Agersew Alemu

**Affiliations:** 1 Department of Immunology and Molecular Biology, School of Biomedical and Laboratory Sciences College of Medicine and Health Sciences, University of Gondar, Gondar, Ethiopia; 2 Department of Clinical Chemistry, School of Biomedical and Laboratory Sciences, College of Medicine and Health Sciences, University of Gondar, Gondar, Ethiopia; 3 Department of Medical Microbiology, School of Biomedical and Laboratory Sciences, College of Medicine and Health Sciences, University of Gondar, Gondar, Ethiopia; 4 Department of Medical Parasitology, School of Biomedical and Laboratory Sciences, College of Medicine and Health Sciences, University of Gondar, Gondar, Ethiopia

**Keywords:** Health care workers, Post exposure prophylaxis, HIV

## Abstract

**Background:**

HIV/AIDS infection in health care facility has become a major health problem. Especially in resource poor setting health care workers are managing huge number of HIV infected patients that made them to be more exposed to HIV infection. This situation makes the use of post exposure prophylaxis for HIV very important. Therefore the aim of the study was to assess knowledge, attitude and practice of health care workers towards post exposure prophylaxis for HIV.

**Methods:**

Cross-sectional study was conducted among 195 health care workers from February 15 to June 20, 2012. Data was collected using self-administered questionnaire and entered and analyzed using SPSS-20 version. Results were summarized in percentages and presented in tables.

**Results:**

Significant proportions of respondents, 72 (36.9%), were found to have inadequate knowledge about post exposure prophylaxis for HIV. However the majority of respondent 147 (75.4%) had good attitude toward the PEP and significant number of the respondents, 66 (33.8%), had been exposed to blood, body fluids, needles or sharp objects once or more times while giving care for patients. Among these exposed, 49 (74.2%) took PEP but the rest 17 (25.7%) didn’t take PEP. From these exposed respondents that took PEP, 23 (46.9%) correctly started taking of PEP at exact initiation time, but the rest started after the recommended initiation time. Among those who took PEP, 39 (79 .6%) completed taking the drug, however 10 (20.4%) didn’t complete the PEP regimen.

**Conclusion:**

As a conclusion, significant proportion of study subjects had less knowledge and practice even though the majority of respondents had favorable attitude towards PEP. Therefore, a formal training for all HCWs regarding PEP for HIV and also establishing a 24 hour accessible formal PEP centre with proper guideline is recommended.

## Background

In order to prevent transmission of pathogens after potential exposure and also to refer for comprehensive management to minimize the risk of infection after potential exposure to HIV, post exposure prophylaxis (PEP) is needed [1]. PEP includes first aid, counseling, risk assessment, relevant laboratory investigations based on the informed consent of the exposed person and source and following the risk assessment, provision of short term of antiretroviral drugs for 28 days, along with follow-up evaluation [2].

Health care workers (HCWs) are persons working in health care setting and they are potentially exposed to infectious materials such as blood, tissue, specific body fluids, medical supplies, equipment or environmental surfaces contaminated with these substances [2]. They are frequently exposed to occupational hazards through per-cutaneous injury such as needle stick or cut with sharps, contact with the mucus membrane of eyes or mouth of an infected person, contact with non intact skin exposed with blood or other potentially infectious body fluids [3].

When we focus on HCWs that are found in developing countries, they are at serious risk of infection from blood borne pathogens like HIV, Hepatitis B and C viruses because of the high prevalence and increased occupational risk of these pathogens in the areas [4,5]. Unsafe practices like careless handling of contaminated needles, unnecessary injections on demand, reuse of inadequately sterilized needles, and improper disposal of hazardous waste (major problem in developing countries) can increase the potential risk of occupational transmission of these blood borne pathogens [6].

Different evidences showed that there is an information gap in the health care setups regarding PEP. For instance a study conducted in London indicated that only 22% of doctors identified all the three drugs that are recommended at that time [7]. A study conducted in Ethiopia, Jimma town, showed that 83.9% of total HCWs had inadequate knowledge about PEP for HIV and among the exposed respondents, 81.6% did not use PEP of whom 33.8% didn’t use PEP because of lack of information [8].

In Gondar, there is no study conducted about PEP for HIV on HCWs. Thus, this study was undertaken to assess knowledge, attitude, and practice about HIV post exposure prophylaxis among health care workers of Gondar University Hospital, Gondar, and Northwest Ethiopia.

## Methods

### Study design and area

Cross sectional study was conducted from February 15 to June 20, 2012 among health care workers of Gondar university hospital. Gondar town is one of the oldest and historical places located 738 km to the North West of Addis Ababa. Gondar University Hospital is a tertiary level referral hospital that serves more than 5 million people in and around Gondar town. It has more than 500 beds with one intensive care unit.

### Sample size and sampling technique

The sample size was determined by using single proportion formula (n = [Z α / 2] ^2^ P (1-p] / d^2^) at 95% confidence interval, where, Z α / 2 = 1.96, P = prevalence of 50% was taken since there is no similar study in the study area and d = 5% of marginal error was taken. Using this calculation, we obtained 384 to be the sample size. Since the exact number of source population of respondent is less than 10,000, we used correction formula of nf = ni / (1 + ni/N) where nf = corrected sample size ni = uncorrected sample size, and N = total number of all the source population [9]. Therefore, (384/ (1 + 384/400 = 195), we obtained sample size of 195.

The total sample size was distributed proportionally across different health professionals involved in this study and the study subjects were selected using simple random sampling technique.

### Data collection

Structured self administered questionnaire having the common sociodemographic characteristics and questions that can assess the levels of their knowledge, attitude and practice towards PEP for HIV was prepared in English version by the research team. Then it was translated into the Amharic, local language of the study area by linguistic professionals. Matching was made on the exact fitness of the two versions. A pretest using the questionnaire was conducted among fifteen percent of the total sample size that is not to be included in the study. The pretest as well as the study was done by trained data collectors and any ambiguous and unsuitable questions were modified after the pretest had been conducted.

### Scoring of knowledge, attitude and practice

Eight questions, with “Yes” (for correct answers) or “No” (for incorrect answers) response, were prepared to assess the knowledge of respondents about PEP for HIV and those respondents who scored greater than or equal to 70% were considered knowledgeable. A seven item question was used to assess participants’ attitude towards PEP for HIV and those who score 70% and above were considered as having good attitude. To assess the practice of respondents’ seven questions were prepared and those who answered “Yes” to more than 70% of the questions were considered as if they are practicing PEP for HIV.

### Data analysis

Data was entered, cleaned and analyzed using SPSS version 20 computer software. Results were summarized in frequencies and percentages and presented in tables.

### Ethical consideration

The study secured ethical clearance from ethical committee of School of Biomedical and Laboratory Science in the University of Gondar .The HCWs were registered to participate in the study only after they obtained explanation about the objectives of the study and also we obtained written consents from study participants. Confidentiality of the study subjects was maintained.

## Results

### Sociodemographic characteristics

A total of 119 (61%) males and 76 (39%) females responded in this study. Most of respondents 134 (68.7%) were in the age group 20 to 30 years. Regarding year of service of HCWs, 83 (42.6%), 71(34%), 12 (9.2%), 23 (10.8%) served for 6 month-2 years, 3-5 years, 6–8 years more than 8 years respectively (Table 1).

**Table 1:** Sociodemographic characteristics of HCWs in Gondar University Hospital, 2012

**Variables**		**N (%)**
Age of respondents	20-30 year	134 (61.0)
	31-40 year	42 (21.5)
	41-50 year	15(7.7)
	>50	4 (2.1)
Sex	Male	119 (61)
	Female	76 (39)
Work experience	6 month- 2 years	83 (42.6)
	3-5 years	71 (36.4)
	6-8 year	18 (9.2)
	>8 year	23 (11.8)
Marital status	Married	53 (27.2)
	Single	135 (69.2)
	Divorced	5 (2.6)
	Widowed	2 (1)
Religion	Orthodox Christian	111 (56.9)
	Protestant Christian	38 (19.5)
	Muslim	42 (21.5)
	Other	4 (2.1)
Profession	Medical Doctor	55 (28.2)
	Nurse	45 (23)
	Lab. Tech.	46 (23.5)
	Health officer	4 (2)
	Anesthetics	4 (2)
	Midwifes	32 (16.4)
	physiotherapy	9 (4.6)
Educational level	Certificate	3 (1.5)
	Diploma	41 (21)
	First Degree	133 (68.2)
	Masters Degree	14 (7.2)
	Specialist	4 (2.1)

### Knowledge level of the HCWs about PEP for HIV

In general, majority, 123 (63.1%), of the participants of the study had adequate knowledge about PEP for HIV. The proportion of respondent who heard about PEP of HIV from formal training was 95 (48.7%). From the study participants 118 (60.5%) answered that PEP for HIV is efficient and 99 (50.7%) knew when to initiate PEP for HIV. One hundred and forty one (72.3%) of the respondents knew the maximum acceptable delay to take PEP for HIV and 142 (72.8%) knew for how long exposed individuals should be on PEP to prevent infection (Table 2).

**Table 2:** Response of HCWs to each question that assess their knowledge about PEP in Gondar University Hospital, 2012

**Knowledge questions**	**Responses**	**Frequency (%)**
Heard about PEP	Yes	181 (92.8)
	No	14 (7.2)
From what source you got the information?	Training	95 (48.7)
	Mass media	10 (5.1)
	Friends	63 (32.3)
	Journals	6 (3.1)
	Other	7 (3.6)
	Multiple answer	14 (7.2)
When do you think PEP should be indicated?	When the source patient is at high risk for HIV	35 (17.9)
	When the patient is known to be HIV positive	54 (27.7)
	When the HIV status of the source is unknown	29 (14.9)
	For any needle stick injury in the work place	30 (15.4)
	Multiple answer	47 (24.1)
What is the maximum delay to take PEP?	24 hour	19 (9.7)
	48 hour	17 (8.7)
	72 hour	141 (72.3)
	12 hour	18 (9.2)
What is the preferable time to take PEP?	Within an hour	99 (50.8)
	After 6 hour of exposure	34 (17.4)
	After 12 hour of exposure	13 (6.7)
	After 72 hour of exposure	49 (25.1)
What is the Effectiveness of PEP?	100%	23 (11.8)
	80-100%	118 (60.5)
	60-70%	45 (23.1)
	30-50%	7 (3.6)
	20-30%	2 (1.0)
What is the length of time to take PEP?	For 28 days	142 (72.8)
	For 40 days	32 (16.4)
	For six moths	18 (9.2)
	For life time	3 (1.5)
Have you attend any training about PEP?	Yes	127 (65.1)
	NO	68 (34.9)
Do you know about the PEP guideline?	Yes	86 (44.1)
	NO	53 (27.2)
	I do not know	56 (28.7)

### Attitude of the HCWs about PEP for HIV

Majority of the respondent, 192 (98.5%) and 172 (88.2%), agreed on the importance of PEP for HIV and the availability of PEP guidelines in the hospital or in their work place. When we assessed the respondents about their belief on PEP for HIV to reduces the likelihood of being infected by HIV after being exposed, 153 (78.5%) of them had strong believe that it can reduce the probability to be infected and also 52 (26.7%) of the respondents agreed that PEP prevent further infection. The believe that PEP may be indicated for any type of sharp object injuries was also assessed among the respondents and it was observed that 57 (29.2%) of the respondents had that believe but the majority, 89 (45.6%), of the study participants did not agree on it and the rest of the study participants 49 (25.1%) were not sure about it. Generally, the attitude of most of the respondents, 147 (75.4%), was good whereas 48 (24.6%) had unfavorable attitude towards PEP for HIV (Table 3)**.**

**Table 3:** Attitude of HCWs about PEP in Gondar University Hospital, 2012

**Questions**		**Frequency**
Do you think PEP is Important?	Yes	192 (98.5)
	NO	3 (1.5)
	I am not shore	-
Do you believe that training of PEP is important for a behavioral change?	Agree	186 (95.4)
	Disagree	9 (4.6)
	Neutral	
Do you think there should be PEP guideline in work areas?	Agree	135 (69.5)
	Disagree	13 (6.7)
	Strongly agree	37 (19)
	No comment	10 (5.1)
Do you believe PEP reduces likelihood of being HIV positive	Yes	153 (78.5)
	No	28 (14.4)
	I am not sure	14 (7.2)
Do you believe PEP to prevent further infection?	Agree	52 (26.7)
	Disagree	117 (60)
	Partially agree	26 (13.3)
How do you see the saying that PEP is indicated for any type of sharp injuries	Agree	57 (29.2)
	Disagree	89 (45.6)
	I am not sure	49 (25.1)
What is your opinion on the believe that PEP is not important if the exposure is not with patient blood of known HIV positive	Agree	30 (15.4)
	Disagree	142 (72.8)
	I am not sure	23 (11.8)

### Practice status of the HCWs towards PEP for HIV

Among all of the respondents, 66/195 (33.8%) were exposed for HIV risky conditions and of these exposed respondents, 49/66 (74.2%) took PEP. However, 17/66 (25.7%) of the exposed respondent did not take PEP. Among the respondents who took PEP, 28/49 (57.1%) reasoned out that they took PEP for their exposure to known HIV positive blood whereas, the remaining 12/49 (24.5%), reasoned out that they became exposed to blood of HIV unknown status. Among all respondents who took PEP, 23(46.9%) correctly started taking of PEP at exact initiation time, but the rest of them start after the recommended initiation time. Furthermore, among those respondents that took PEP, 39/49 (79.5%) had completed taking PEP correctly, but the rest 10/49 (20.4%) had failed to complete. The reasons for the discontinuity of taking the PEP was found to be fear of its efficacy and the adverse effects 5/10 (50%), 3/10 (30%) respectively (Table 4).

**Table 4:** Practice of PEP for HIV among HCW in Gondar University Hospital, 2012

**Questions**	**Responses**	**Frequency**
Ever been exposed to HIV risky conditions	Yes	66 (33.8)
	No	119 (61)
	I do not remember	10 (5.2)
took PEP after exposure	Yes	49 (74.2)
	No	17 (25.7)
The reason respondent to took the PEP	Exposure to blood from known HIV positive patients.	28 (57.1)
	Exposure to blood from patient whose HIV status is unknown	12 (24.5)
	Injury from any sharp objects	6 (12.2)
	Contact with patient body fluids	3 (6.1)
The time to start taking the PEP	With in 1 hour	23 (46.9)
	After 2–6 hrs of exposure	15 (30.6)
	After 6–10 hrs of exposure	10 (20.4)
	After 72 hrs	1 (2)
A period of time that a respondent take PEP	3 days	3 (6.1)
	15 days	7 (14.3)
	28 days	39 (79.5)
completed the prescribed drug of PEP	Yes	39 (79.6)
	No	10 (20.4)
reason for discontinuation of the drug	Fear of adverse effects	3 (30)
	Assuming that it was enough	2 ( 20)
	Assuming that the drug was not effective	5 (50)

## Discussion

This study assessed the knowledge, attitude and practice towards PEP for HIV among HCWs who were directly involved in care of patients in Gondar University Hospital which is located northwest of Ethiopia.

In the present study, among all study participants 92.8% have heard about PEP for HIV. When we compare it with other study which was conducted in a tertiary hospital in Nigeria (97%), it was found that less percentage of the study participants in the present study had been found who heard about PEP [10].

Regarding when to start PEP for HIV, in the present study 50.8% of the total respondents responded stating PEP should be taken within one hour which is higher than other findings from study conducted in Mulago Hospital in Uganda with only 22.3% being sure it should be started within an hour of exposure [11]. In another study among interns, only 31.6% of respondents stated the exact time when to initiate PEP which is also lower than our report [12]. However when we observe a study conducted in Mumbay it showed that 64% of the respondent correctly stated when to start PEP in which it is greater than the present study [13]. The difference might be because of the difference on the level of awareness among the different populations. The proportion of knowledgeable participants on when to start PEP for HIV is still low because only half the respondents stated it correctly. Therefore, if the remaining 50% of the respondents exposed for HIV risky conditions, they might took PEP after very long period of time so that they will be important sources of transmitting HIV [14].

A study conducted in Zimbabwe showed that 65% of the respondents scored less than 50% of the questions regarding knowledge which was regarded as poor knowledge [15]. In the present study the percentage of the respondents with poor knowledge is 36.9% which indicated that it is better than the findings of the study conducted in Zimbabwe. However, this level of poor knowledge cannot be considered low.

In the present study, from195 subjects, 66 (33.8%) of the respondents have been exposed for HIV risky conditions. This finding is less than the result found in a study conducted in south India in which 74.5% of respondents were exposed [16]. However, the number of HCWs that have ever been exposed to HIV risky conditions in the present study could not be considered as low since in Italy a study indicated only 11.3% of occupational exposure which is lower than the present study [17]. Generally the difference between the present study and the others might be due to the difference in the setting.

Even though 74.2% of the exposed respondents took PEP for HIV in this study, only 60.9% of these respondents were able to complete the regimen of the drug which requires 28 days. This finding was in agreement with other study conducted in Dar es Salaam in which they showed that 40% of the respondents failed to use PEP for the full length of time prescribed [18]. However, study conducted in Gujarat showed that their respondents had better practice in this regard than our study participants in which more than 94% were able to complete the regimen [19]. This fact alerts that the practice of PEP for HIV in the study area needs improvement.

Reasons for the observed difference of findings between different research results might be due to the difference in the level of awareness between the different population, economic difference of the study population and time difference of the studies.

## Conclusion

In general, the findings of this study revealed the gap that knowledge as well as practice of HCWs towards PEP for HIV is inadequate. Even though many of the HCWs had HIV risky exposure, the number of HCWs that were exposed but did not take the PEP for HIV cannot be considered as low. Therefore, a formal training for all HCWs regarding PEP for HIV should be provided to improve their knowledge and also establishing a 24 hour accessible formal PEP centre with proper guideline is recommended so that their practice towards utilization of PEP can be improved.

Besides, new strategies must be developed to reduce the risk of occupational exposure in health care facilities.

## Competing interests

The authors declare that they have no competing interests.

## Authors’ contributions

BM initiates and design the study, drafted the manuscript. WB involved in data analysis and manuscript review. SK, MB, GT involved in data collection and analysis. ZA and AA participated in data analysis and manuscript review. All authors have read and approved the final manuscript.

## Pre-publication history

The pre-publication history for this paper can be accessed here:

http://www.biomedcentral.com/1471-2458/13/508/prepub

## References

[B1] Sharma A Marfatiya YA Ghiya R Post-exposure prophylaxis for HIV Indian J Sex Trans Dis and AIDS 2007 28 2.61 68

[B2] Ministry of Health and Family Welfare Government of India National AIDS Control Organization: Management of Occupational exposure including Post exposure prophylaxis for HIV 2009 NewDelhi: NACO

[B3] Ministry of Health and Family Welfare, Government of India National AIDS Control Organization Antiretroviral Therapy Guidelines for HIV-infected Adults and Adolescents including.post-exposure prophylaxisnone 2008 New Delhi: NACO

[B4] Simonsen L Kane A Lloyd J Zaffran M Kane M Unsafe injections in the developing world and transmission of blood borne pathogens: a review Bull World Health Organ 1999 77 789 800 10593026 PMC2557743

[B5] Kane A Lloyd J Kane M Transmission of hepatitis B, hepatitis C and HIV through unsafe injections in the developing world Bull World Health Organ 1999 77 801 807 10593027 PMC2557740

[B6] Charles SM Richard DP Janine J Risk to health care workers in developing countries N Engl J Med 2001 345 538 541 10.1056/NEJM200108163450711 11519511

[B7] Khan AZ Ducan KM Escotet X Miles WEA do we need to improve awareness about HIV post exposure prophylaxis? Ann R Coll Surg Engl 2002 84 72 73 11892732 PMC2503757

[B8] Bosena T Chernet H assessment of HIV exposure prophylaxis among health workers of governmental health institute in jimma zone, Oromia Region, south west Ethiopia Ethiop J.health sci 2010 1 55 64 10.4314/ejhs.v20i1.69429 PMC3275901 22434961

[B9] Thrusfield M Veterinary Epidemiology 1995 London: Blackwell Science

[B10] Owolabi RS Alabi P Ajayi S Daniel O Ogundiran A Akande TM Onafowokan T Knowledge and Practice of Post Exposure Prophylaxis (PEP) against HIV Infection among Health Care Providers in a Tertiary Hospital in Nigeria JIAPAC 2012 11 3 179 183 21511981 10.1177/1545109711401409

[B11] Alenyo R Fualal J Jombwe JJ Knowledge attitude and practice of staffs towards post exposure prophylaxes for HIV infection at Mulango hospital in Uganda East and central African J Surgery 2009 14 2 99 102

[B12] Chacko J Isaac R Percutaneous injuries among medical interns and their knowledge and practice of post exposure prophylaxis for HIV Indian J Public Health 2007 51 127 129 18240478

[B13] Chogle NL Chogle MN Divatia JV Dasgupta D Awareness of post-exposure prophylaxis guidelines against occupational exposure to HIV in a Mumbai hospital M Natl Med J India 2002 5 2 69 72 12044118

[B14] Varghese GM Abraham OC Mathai D Post-exposure prophylaxis for blood borne viral infections in healthcare workers Postgrad Med J 2003 79 324 328 10.1136/pmj.79.932.324 12840120 PMC1742734

[B15] Monera T Ncube P Assessment of Knowledge, Attitude and practice of health care workers on Occupational HIV post exposure prophylaxis at Zimbabwean referral hospital J Int AIDS Soc 2012 15 4

[B16] Tetali S Choudhury PL Occupational exposure to sharps and splash: Risk among health care Providers in three tertiary care hospital in south india Indian J Occup Environ Med 2006 10 1 35 40 10.4103/0019-5278.22894

[B17] Bandolier E Occupational exposure to hospital employees in Italian hospitals over 5.5 years 2003 London: AstraZeneca group

[B18] Chagani MM Manji KP Manji MP Sheriff FG Healthcare workers’ knowledge, attitudes, practices on post exposure prophylaxis for HIV in Dar es Salaam Tanzania Med J 2011 25 2 33 38

[B19] Shevkani M Kavina B Kumar P Purohit H Nihalani U Shah A An overview of post exposure prophylaxis for HIV in health care personals: Gujarat scenario Indian j sex transm Dis 2011 32 1 9 13 10.4103/0253-7184.81247 21799569 PMC3139298

